# Heparin-Binding Epidermal Growth Factor-like Growth Factor/Diphtheria Toxin Receptor in Normal and Neoplastic Hematopoiesis

**DOI:** 10.3390/toxins5061180

**Published:** 2013-06-18

**Authors:** Fabrizio Vinante, Antonella Rigo

**Affiliations:** Department of Medicine, Section of Hematology, Cancer Research & Cell Biology Laboratory, University of Verona, Verona, Italy; E-Mail: antonella.rigo@univr.it

**Keywords:** HB-EGF, GPCR, EGFR/ErbB/HER, Hematopoiesis, Leukemia, Myeloma

## Abstract

Heparin-binding EGF-like growth factor (HB-EGF) belongs to the EGF family of growth factors. It is biologically active either as a molecule anchored to the membrane or as a soluble form released by proteolytic cleavage of the extracellular domain. HB-EGF is involved in relevant physiological and pathological processes spanning from proliferation and apoptosis to morphogenesis. We outline here the main activities of HB-EGF in connection with normal or neoplastic differentiative or proliferative events taking place primitively in the hematopoietic microenvironment.

## 1. HB-EGF

**Figure 1:**
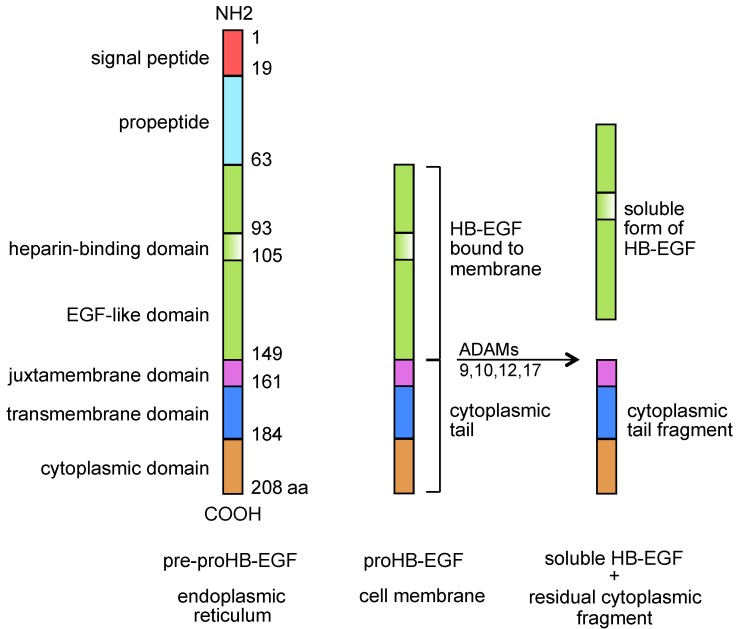
Basic structure, functional domains, and processing of HB-EGF.

Heparin-binding epidermal growth factor-like growth factor (HB-EGF, Figure 1) was first described by Higashiyama and Coll [1,2], in 1991/92, as a heavily glycosylated EGF family member of approximately 22 kD that was released by macrophage-like U-937 cell line, showing heparin affinity and eliciting mitogenic activities in BALB-3T3 fibroblasts and smooth muscle cells, but not in endothelial cells. As a member of the EGF family, it bound to EGF receptors on A-431 epidermoid carcinoma cells and smooth muscle cells, and was a more potent mitogen for smooth muscle cells than EGF. HB-EGF was also described as expressed in cultured human macrophages and involved in macrophage-mediated cellular proliferation. It turned out that membrane-anchored HB-EGF and the sole receptor for diphtheria toxin were one and the same molecule [3,4,5]. Diphtheria toxin binds to HB-EGF [5] and after internalization inhibits protein synthesis, an event that usually triggers apoptotic death [6]. It is now recognized that HB-EGF is expressed in a wide range of cell types, including monocytes/macrophages [1], CD4+ lymphocytes [7,8], neutrophils [9], eosinophils [10], myeloid leukemia blasts [11], myeloma cells [12], vascular smooth muscle cells [13], endothelial [14] and normal [15], or neoplastic [16,17,18] epithelial cells. HB-EGF binds to EGFR/ErbB1/HER1 and ErbB4/HER4 [1,2,19] (Table 1) eliciting different biological responses [17,19]. Membrane-anchored HB-EGF functions as a cell-to-cell adhesion protein [20] that exerts mainly inhibitory juxtacrine activities involved in cell cycle arrest and growth inhibition [21] as well as resistance to apoptosis [22,23]. HB-EGF may also be cleaved and released from cell upon stimulation with a variety of molecules capable of activating integral membrane metalloproteases with a disintegrin domain (ADAMs) 9, 10, 12, and 17 that can be viewed as activators of the paracrine form of HB-EGF [24,25,26,27,28,29]. Soluble HB-EGF acts variously on sensitive bystander cells, including mitogenic and chemotactic effects on monocytes/macrophages [1], fibroblasts [7], and SMC [30,31], chemotaxis on endothelial cells [30], astrocytes [32], and growth activity for normal and neoplastic epithelial cells [8,15]. The shedding process generates a kind of autocrine mitogenic signal on the releasing cells themselves via nuclear translocation of either full-length proHB-EGF [33] or its truncated cytoplasmic tail [34] (Figure 1). HB-EGF is involved in orchestrating inflammation [18], atheromatous plaque progression [8], and wound healing [7,8,35,36]. It participates in stromal proliferation following decidualization [37], and in autocrine-paracrine loops, which are active in a number of epithelial neoplasias [8,18], by promoting tumor growth as a direct mitogen for neoplastic cells [8,18,38,39] or a microenvironment modifier by inducing angiogenesis [7,8,18,31,39,40,41] and recruiting a variety of cells [7,8,18,42,43,44]. Either membrane-anchored or soluble HB-EGF has a role in directing the proliferation and differentiation of stromal stem cells [45] and in epithelial-to-mesenchymal transition [46]. Due to its heparin-binding domain, a unique property of the EGF receptor family ligands, HB-EGF is able to bind to heparan-sulphate proteoglycans, which may further modulate its biological activity [17]. Such heterogeneous and proteiform activities make sense based on the evolutionary and functional history of this growth factor. A molecule involved in proliferation [1], apoptosis [21,23], cell migration [42,43], differentiation [45], morphogenesis, and development [47,48,49,50,51], HB-EGF emerged as a microenvironment organizer, a factor contributing to, and acting in, niches where normal stem and progenitor cells are maintained and ordinately released into differentiation and where oncogenic processes take place and keep developing.

**Table 1:** EGF family receptors and ligands.

EGFR/ErbB1/HER1	ErbB2/HER2	ErbB3/HER3	ErbB4/HER4
HB-EGF		NRG1/2	HB-EGF
EGF			NRG1/2/3/4
TGF-α			BTC
AR			EPR
BTC			
EPR			
ErbB/HER homo- and heterodimers activated by HB-EGF 1/1 - 1/2 - 1/3 - 1/4 - 2/4 - 3/4 - 4/4			

TGF-α, transforming growth factor-α; AR, amphiregulin; BTC, betacellulin; EPR, epiregulin; NRG1/4, neuregulin-1/4.

## 2. EGF Family Members, Cognate Receptors, and Layers of Regulation

The members of the EGF family include four groups of growth factors binding to specific cognate receptors (EGFR/ErbB1/HER1, ErbB2/HER2 to ErbB4/HER4). Upon ligand binding, the receptors associate to form a variety of di/oligomeric complexes acting as intrinsic tyrosine kinases [35,36,37]. As shown in Table 1, EGF ligands bind to specific receptors with a degree of crossreactivity also due to the assortment of these complexes. Soluble and membrane-anchored HB-EGF bind to, and activate, EGFR [1,4], ErbB4 [19,55], and complexes of these receptors [53,54,56]. HB-EGF also binds to heparan sulfate proteoglycans [55,57], and N-arginine of dibasic convertase [42]. ErbB receptors are expressed within numerous tissues and systems where they may mediate different activities depending on the ligand, its sterical organization (membrane-anchored or soluble receptor), and its receptor affinity. For example, soluble HB-EGF ligation of EGFR mediates strong proliferative effects [38,39], while the ligation of ErbB4 mediates chemotactic responses [19]. Instead, through the same receptors membrane-bound HB-EGF may mediate growth inhibition and functional responses [21,22,23]. Therefore there is a level of regulation, based on the molecule topology, which leads to biochemical differences and different resulting activities [58]. A major difference is that membrane-anchored HB-EGF is believed to induce extended polymerization of ErbB receptors, whereas soluble HB-EGF probably induces smaller receptor complexes, usually in form of homo/heterodimers or small oligomers. Extended complexes imply a variety of heterogeneous cell-to-cell interactions including coreceptors activities, and sending, usually, inhibitory signals. Smaller complexes imply selective receptor stimulation and send activatory signals [58]. Finally, heparan sulfate proteoglycans on the cell membrane may regulate the activity of HB-EGF [57] and the expression of N-arginine of dibasic convertase enhances the migration induced by HB-EGF via EGFR [42]. In other words, a further source of cellular signaling is the transmembrane/cytoplasmic fragment of HB-EGF, either as part of full-length internalized proHB-EGF [33,59] or as truncated transmembrane/cytoplasmic fragment remaining after the ADAM-dependent release of the ectodomain portion [34,59]. Under stimulus this transmembrane/cytoplasmic fragment translocates to the nucleus and binds to transcriptional cyclin A repressor promyelocytic leukemia zinc finger protein [34] or to transcriptional cyclin D2 repressor Bcl6 [60] promoting S-phase entry. Also, the phosphorylation of the cytoplasmic domain of HB-EGF due to external stimuli plays a role in HB-EGF-related tumorigenesis [61]. Therefore the cells and enzymes available in the microenvironment add a further layer of local regulation to HB-EGF biological activities. Enhanced expression, constitutively active mutant forms, autocrine activation of the ErbB receptors, and increased HB-EGF production are observed in human cancers and may be associated with poor outcomes. Therapeutic strategies targeting EGFR, ErbB2 [62] or HB-EGF [63,64,65] have been suggested or even used in medical practice.

## 3. HB-EGF-Dependent EGFR Phosphorylation and Transduction Pathways

HB-EGF induces receptor association, intrinsic tyrosine kinase-dependent autophosphorylation of specific tyrosine residues that serve as docking sites for intracellular signaling molecules, and activates a signaling cascade to nucleus followed by cell proliferation [1], differentiation [45] or chemotaxis [42] depending on the receptors that are involved. Upon HB-EGF, for example, EGFR activates intrinsic protein tyrosine kinase activities that phosphorylate specific tyrosine residues within the cytoplasmic domain [66]. By performing mass spectrometry analysis of trypsin digested peptides on HeLa cells we observed that HB-EGF induces EGFR phosphorylation at tyrosines 992, 1045, 1068, 1086, 1148, 1173, and at serine 1142. In contrast, there is no phosphorylation of tyrosine 1045, little phosphorylation of serine 1142, and as a rule, HB-EGF-induced HER1 phosphorylation shows a pattern somewhat different from that induced by other EGFR ligands [18,67]. Tyrosines 1068 and 1173 are two major sites of autophosphorylation that autophosphorylated, to very different degrees, following HB-EGF [18]. Autophosphorylation of EGFR tyrosines 1068 and 1173 is followed by the activation of Ras, MEK, and ERK1/2 pathways [68]. For example, following stimulation of HeLa cells with HB-EGF, ERK1/2 phosphorilates at threonine 185 and tyrosine 187 [18,67].

## 4. GPCR-Dependent Transactivation of EGFR

Ligation of G-protein (heterotrimeric guanine nucleotide-binding protein)-coupled receptors (GPCRs) leads to ADAM-dependent cleavage [69] of membrane-anchored HB-EGF into a soluble form of HB-EGF [24,70] capable of activating ErbB receptors on the same cell or on bystander cells [71,72]. Indeed, the transactivation of EGFR is a general function of GPCR signaling [18,24,67,73,74,75,76,77], which has been described in diverse cell types including fibroblasts, keratinocytes, astrocytes and smooth muscle cells [18,58,73]. Deregulated GPCR-dependent release of HB-EGF has been associated with hyperplastic tissue abnormalities [78]. Critical mitogenic effects through extracellular transactivation of EGFR follow to stimulation with GPCRs ligands including endothelin-1, lysophosphatidic acid [50], angiotensin-II [73] or CXC chemokine ligand 12 (CXCL12, stromal cell-derived factor-1) [18] via ADAMs 10, 12, or 17 depending on the ligand, GPCR, and cell type [24,25,26,27,28,29,79]. We demonstrated that CXCL12 transactivates EGFR through ectodomain shedding of HB-EGF [18,67]. CXCL12 binds to GPCRs CXC receptor 4 and 7 (CXCR4 and CXCR7) [80], and activates ADAM 10 at least in macrophages [18,67,81,82,83] and might increase the release of ADAM 17 [84]. Interestingly, ADAM 10 mediates also the cleavage of Notch and its ligand Delta [85]. The CXCL12-dependent HB-EGF transactivation mechanism is relevant to hematopoietic differentiation as well as leukemia progression [86]. Besides its role in development [87], CXCL12 regulates important hematopoietic functions, induces cell adhesion and chemotaxis, and coordinates the circulation of hematopoietic stem cells, lymphocytes and monocytes/macrophages [86,88,89,90,91]. Stromal and endothelial cells constitutively express CXCL12 in bone marrow, lymph nodes, liver, lung and skin [92]. CXCL12 may induce mitotic signals, favor tissue invasion, and contribute to the development of a microenvironment infiltrated by M2-polarized macrophages that support cell survival [91,93]. Cancer cells usually express functional receptors for CXCL12 [67,89,90,94] and some cancers constitutively express CXCL12 [18,95]. 

## 5. GPCR-Dependent Transinhibition of EGFR

By binding to its receptors, CXCL12 may transactivate EGFR. As described above, for example, the ligation of CXCR4 activates membrane ADAMs that release HB-EGF from cell membrane, resulting in its binding to EGFR [24,69,70,71,72]. This induces a signaling cascade that is involved in pivotal phenomena such as proliferation or differentiation as well as morphogenesis depending on the microenvironmental context [18,50,73]. Usually, the transactivation occurs in a paracrine manner. In EGFR/GPCR double-positive cells, however, a transactivation along intracytoplasmic pathways has been reported [96] and shown to be important in breast cancer [97] and myeloma progression [98]. Furthermore, we provided evidence that in EGFR/CXCR4 double-positive cells CXCL12 may transinhibit EGFR [67]. Downstream of CXCR4, CXCL12 elicits signals via both G protein-dependent and β-arrestin-dependent pathways responsible for inducing rapid and late waves, respectively, of ERK1/2 phosphorylation [99,100,101]. In HeLa and 5637 cancer cell lines, CXCL12 elicits an intracytoplasmic G-protein-dependent calmodulin/calcineurin pathway leading to a delayed autophosphorylation peak of EGFR that is reverted by later β-arrestin activation. The synthetic [N33A] CXCL12 [102] that activates preferentially G protein-dependent pathways and fails to activate β-arrestin-dependent ERK1/2 phosphorylation downstream of CXCR4 [67] can completely block EGFR phosphorylation [67]. Therefore a EGFR/CXCR4 double-positive cell may shed HB-EGF to tyransactivate bystander EGFR-positive cells [24,69,70,71,72], transiently delay the activation of its own EGFR [67] and enter into S-phase due to nuclear translocation of the cytoplasmic tail of HB-EGF [34]. This process can be expected to strengthen a gradient of cell proliferation along differentiation pathways functional to the maintenance of hematopoietic niches. 

## 6. Factors Inducing HB-EGF in Hematopoietic Cells

A great variety of human hematological cell lines except for the B-derived ones, and blasts from a majority of acute myeloid leukemia cases express and release a fully functional HB-EGF molecule. These cells express HB-EGF mRNA, are sensitive to the cytolytic effect of diphtheria toxin exposure, and release soluble HB-EGF that exerts a strong proliferative effect on BALB/c-3T3 cells [11]. The expression of HB-EGF mRNA in these cells is strongly modified by factors relevant to the biology of hematopoietic cells at different stages of differentiation. DMSO, PMA, ATRA, IFN**γ**, 1α,25-(OH)2D3, and TNFα, are known to induce biological effects such as proliferation or differentiation in normal or neoplastic cells of different hematopoietic lineage. PMA, DMSO and, more interestingly, TNFα, 1α,25-(OH)2D3, and especially ATRA, and costimulation with TNFα and ATRA induce an increase in transcripts for HB-EGF in either cell lines or *ex vivo* cells [9,11]. GM-CSF and CXCL12 also induce HB-EGF in a number of human cell lines, in human monocytes/macrophages, neutrophils, and *ex vivo* cancer cells, including acute leukemia cells [9,11,18]. For example, GM-CSF induces HB-EGF mRNA and acquisition of sensitivity to diphtheria toxin in neutrophils and acute myeloid leukemia cells that were previously HB-EGF-negative [9,11]. In HB-EGF promoter, putative binding sites for NF-kB, AP1, SP1, SP3 have been identified [103]. In addition, it has been shown that HB-EGF can be induced through Ras pathway activation [104,105]. TNFα has been reported to mobilize NF-kB [106]; the receptors for vitamins A and D, including 1α,25-(OH)2D3, recognize common response elements containing the AP1 site [107]; by binding to the β-subunit of its receptor, GM-CSF activates Ras and Raf-1 and the MAP kinase pathway [108]. Therefore, TNFα , ATRA, 1α,25-(OH)2D3, GM-CSF, and CXCL12 have the HB-EGF gene as a downstream target [9,11]. HB-EGF is intensely expressed along the hematopoietic cell differentiation by myeloid [11] and lymphoid progenitors too. Outside the hematopoietic system, there is convincing evidence that HB-EGF is involved in developmental tasks. Pancreatic and duodenal homeobox-1 is also a direct regulator of HB-EGF [109]. The Wilms’ tumor gene contributes to the regulation of the EGF family ligands during nephrogenesis [110]. Direct interaction between MyoD and the HB-EGF promoter is transiently found during skeletal muscle cell differentiation and the membrane form of HB-EGF is expressed preferentially in myotubes [48].

## 7. HB-EGF in Hematopoietic Microenvironment

**Figure 2:**
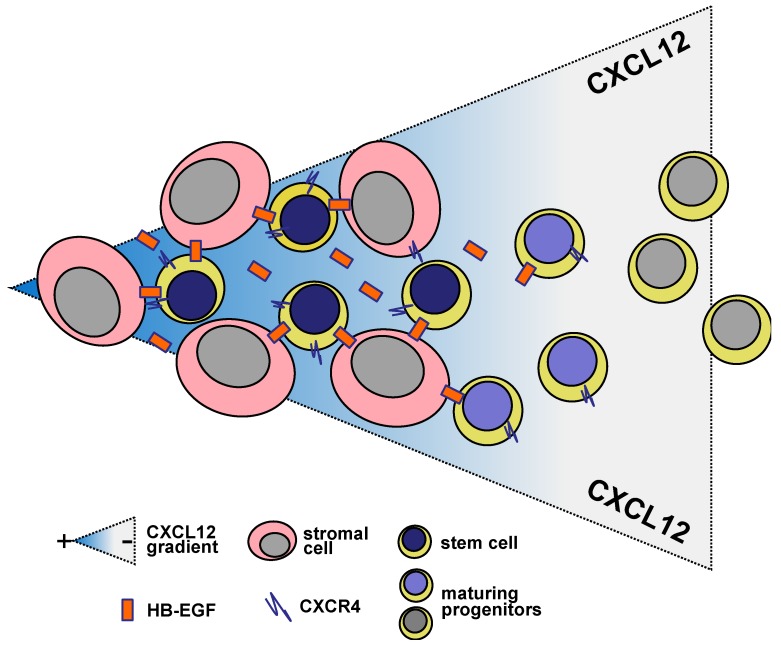
HB-EGF network relevant to hematopoietic niche. Cell-to-cell interactions and gradients of cell-shed factors, including CXCL12 and HB-EGF, lock both hematopoietic and stromal stem cells in the hematopoietic niche where they support each other to keep surviving as undifferentiated cells. Hematopoietic differentiation implies both asymmetric hematopoietic stem cell mitosis and maturing progenitor escape from the niche microenvironment partly due to receptor modulation.

HB-EGF plays a role in regulating the differentiation of stromal stem cells [45] and the proliferation of hematopoietic maturing cells, themselves a source of HB-EGF [9,11]. However, to describe the role of HB-EGF, it is important to stand back and take a broad view of how hematopoietic developmental tasks fit into the organization of what we commonly call the hematopoietic bone marrow microenvironment. Hematopoietic differentiation is characterized by a number of quite well recognized stages. At each differentiative stage, immature cells are present with progressively restricted potential towards the differentiation into blood cells. The differentiation cascade is formed by hematopoietic stem cells and hematopoietic progenitors from which all lineages of blood originate. Hematopoietic stem and progenitor cells seem to be nurtured in sites or niches where cell-to-cell interaction regulate the output of appropriate numbers of blood cells by ensuring hematopoietic stem cell survival and self-renewal [111,112]. The specific hematopoietic stem cell niche allows stem cells to proliferate, prevents them from differentiating, preserving their differentiation potential, and keeps them in place [45,112]. After leaving the stem cell niche, cells can differentiate. Though a variety of cell types have been involved in promoting this regulatory microenvironment [113], CXCL12-expressing reticular cells seem to play a fundamental role [114]. The microenvironment is dynamic and the number of niche cells can correlate with the number of hematopoietic stem cells [115]. The turnover and persistence of the niche is assured by CXCR4, the primary receptor for CXCL12 [80], which plays a pivotal role in maintaining hematopoietic stem cells, early B cell precursors and plasma cells in strict contact with the above-mentioned CXCL12-abundant reticular cells [92,93,114,116,117]. Though hematopoietic stem cells are slowly cycling and some are dormant [118,119,120], lineage-restricted progenitors are cycling actively [121]. CXCL12-abundant reticular cells maintain blood progenitor cells in a proliferative state. Signals that regulate differentially the proliferation of hematopoietic precursors may be elicited by TGF-β [122], angiopoietin-1 [123], Wnt [124], and HB-EGF [9,11,45,125]. Other proteins such as those belonging to Notch family may play a role, which seems to be less relevant than that played by the CXCL2/CXCR4 axis [126]. CXCL12-abundant reticular cells produce hematopoietic cytokines, CXCL12 and SCF [127]. As adipo-osteogenic progenitors, they may differentiate into mature cells that produce protein for bone formation or store energy [45]. Interestingly, cortical thymic epithelial cell compartment is required for early stages of T cell development in the thymus [128]. Cortical thymic epithelial cells express CXCL12 and the Notch ligand Delta-like 4 [129], thus providing signals for the attraction, survival and differentiation for T lymphocyte progenitors [130]. CXCL12-abundant reticular cells release CXCL12 that is able to cleave HB-EGF from bystander cells [24,70,71,72] that in turn inhibits CXCL12-abundant reticular cell maturation [45,67]. Due to their CXCR4 expression [86], either normal hematopoietic or leukemic cells share a preferential homing into the bone marrow, namely into niches with CXCL12-rich ancillary stromal cells [45,92]. There, CXCL12 transactivates EGFR [18] that is mainly expressed by stromal cells and stem cells [45]. Finally, the release of the ectodomain fragment of HB-EGF transactivates EGFR in bystander cells but leads to intracellular transinhibition of EGFR [67] as well as nuclear translocation of the transmembrane/cytoplasmic proHB-EGF fragment, which promotes cell cycling [34,58,59,60]. Therefore hematopoietic progenitor cells increase their proliferative potential entering the differentiation process by the combined effect of CXCL12-dependent shedding of HB-EGF and the intracellular release of HB-EGF transmembrane/cytoplasmic tail to translocate to nucleus to generate mitogenic signals [34]. The regulation of the tyrosine kinase activity of EGFR associated with the activation of cell cycling via direct nuclear factor mobilization further contributes a gradient of cell proliferation and differentiation functional to a flexible and dynamic maintenance of hematopoietic niches. Through chemotaxis and EGFR transactivation, CXCL12 promotes the shaping of niches where stromal and hematopoietic stem cells strictly interact in order to maintain the hematopoietic function (Figure 2).

## 8. CXCL12- and HB-EGF-Related Loops in Hematopoiesis

HB-EGF may be transcriptionally and/or functionally regulated by factors such as TNFα, ATRA, 1α,25-(OH)2D3, GM-CSF, and CXCL12 [9,11,18] that are important for effective and coordinate hematopoiesis. Endothelial and stromal cells in bone marrow constitutively express CXCL12 [92] that induces cytokines that share protean activities involving cell growth and differentiation [22,39,86,87,88,89,90,91,93]. The axis CXCL12/CXCR4 (and possibly CXCL12/CXCR7) may be thought of as a node connecting numerous loops to the highly complex loops involving the EGF family and its receptors, namely HB-EGF/EGFR [53]. In the context of the hematopoietic system, the crosstalk between CXCL12/CXCR4 and HB-EGF/EGFR contributes to the balance between cellular responses of differentiation and self-renewal that depend on the stimulation of EGFR [18,131]. Soluble HB-EGF inhibits the multilineage differentiation of stromal stem cells maintaining them open to proliferation [45] and available for cell-to-cell contact with hematopoietic stem cells in the specialized niches. In contrast, proliferation of hematopoietic progenitors changes from maximal to minimal according to the decreasing gradient of CXCL12 away from the niches. In the niches the balance is tipped preferentially to a self renewal-type of regulation in which the stromal environment participates actively [18,45,67,132,133]. Near the niches hematopoietic progenitors proliferate actively and start their differentiative journey. The hematopoietic microenvironment, therefore, is at the crossroad of regulated self-renewal and oncogenesis and links typically oncogenes and inflammation [134]. Interestingly, terminally differentiated neutrophils, monocytes/macrophages and lymphocytes may also coexpress CXCR4 and HB-EGF [9,11,18]. These cells are sensitive to CXCL12, upon which they release HB-EGF, share aptness to infiltrate normal tissues and cancers and to act either inducing niches supporting cancer stem cells as well as suppressing or fostering immunity. These infiltrating cells seem to reproduce a cancer/stromal microenvironment supporting cancer stem cells somehow similar to the hematopoietic environment. It seems indeed that oncogenesis is primarily coupled to niches [113]. This has suggested a role for the therapeutic use of inhibitors of the CXCL12/CXCR4 [135] and HB-EGF/EGFR [62,63,64,65] axes in the clinical setting, namely targeted to eradicate cancer stem cells that survive in the niches [136].

## 9. HB-EGF in Leukemia

Leukemia encompasses a heterogeneous group of hematological malignancies characterized by a growth of immature (acute leukemias) or variously maturing (chronic leukemias) cells within the bone marrow. Clinical manifestations of acute leukemia include leukocytosis and deficient normal hematopoietic function due to leukemic cells infiltrating the bone marrow [137,138]. Chronic leukemias, including myeloproliferative and lymphoproliferative disorders, show progressive leukocytosis due to accumulation of more or less mature circulating blood cells and deficient hematopoietic function as a late event. They may change their biology and undergo progression to more rapidly evolving conditions, including disorders strictly resembling acute leukemia [139,140]. Perhaps it is not surprising that leukemia might develop by taking over hematopoietic stem cell niches. The relevant role played by ancillary cells in normal hematopoiesis and some emerging models suggest a role for bone marrow microenvironment in disease maintenance and resistance as well as in leukemia initiation and progression [113]. Though the predominant views have been developed in the setting of epithelial cancers, there are data supporting that leukemic cell survival and proliferation may be fostered by niche specific signaling and/or that leukemic cells may modify the microenvironment in order to ease their progression and/or that disruption of signaling between hematopoietic stem cells and their niches promote cancer initiation [113]. A number of cells and factors that govern cell-to-cell interactions and the processes in the bone marrow niches have emerged. CXCL12 [67,114,127] and HB-EGF via its dual signaling [11,18,45,125,141] play a role in remodeling hematopoietic niches allowing for stem cell maintenance and regulated differentiation *vs.* proliferation of hematopoietic progenitors. Most leukemias overexpress HB-EGF [11,63], CXCR4 [136,142], and even CXCL12 [143]. Leukemia-involved cytokines such as TNFα [144,145] and GM-CSF [9,11], or hematopoietic differentiation-related factors such as ATRA and 1α,25-(OH)2D3 [11], have the HB-EGF gene as a downstream target and strongly increase HB-EGF expression [9,11]. Chronic myeloid leukemia cells express the chimeric protein BCR/ABL related to the (9;22) translocation, which plays a relevant pathogenetic role in this leukemia [146]. HB-EGF is one of the molecules transcriptionally induced by the chimeric protein BCR/ABL through activation of the Ras and MAP kinase pathways. Therefore the same factors that play a central role in the hematopoietic niche are involved in leukemia biology [136]. The hematopoietic niche is remodeled by these factors including HB-EGF to represent a protective microenvironment orchestrating a great deal of stimuli favoring leukemia initiation, progression, post-treatment persistence as minimal residual disease, and relapse. 

## 10. HB-EGF in Multiple Myeloma

Multiple myeloma is another malignancy that grows within the bone marrow as a clonal expansion of plasma cells. Over unpredictable time periods, the disease leads to osteolytic bone lesions, hypercalcemia, insufficient haematopoietic function, and anomalous angiogenesis [147,148]. Multiple myeloma is preceded by an asymptomatic premalignant phase associated with bone marrow alterations suggesting microenvironment defects that favor the survival of plasma cells. It seems that additional bone marrow changes are needed for overt myeloma to develop [149]. Myeloma cells take over hematopoietic stem cell niches in bone marrow. A number of molecules relevant to normal haematopoiesis play a part in the biology of myeloma cells such as IL-6, VEGF, osteopontin, angiopoietin-1, Notch, Wnt, Hedgehog, and DKK1 protein [12,149,150,151,152]. Moreover, both homing and survival of myeloma cells largely depends on the CXCL12/CXCR4 axis [152,153]. CXCR4 has been targeted in the clinical setting of multiple myeloma with promising results [154]. EGF-family members such as NRG1, AR, and HB-EGF, which are expressed by cells from hematopoietic niches, can bind to heparan-sulphate proteoglycans on myeloma cells to promote myeloma growth [12,98,149,150,155]. The crosstalk between CXCL12/CXCR4 and HB-EGF/EGFR contributes in this case a direct proliferative stimulus to myeloma cells. However, the primary activity of these loops assures the survival of myeloma stem cells or anything similar inside the bone marrow niches [4,20,156]. In such a protective environment, mainly depending on cell-to-cell interactions based on fundamental players like CXCL12, Notch, and HB-EGF [152], myeloma stem cells can persist as minimal disease surviving treatment to relapse eventually as overt myeloma. EGF-signaling has been proposed as a promising target for myeloma treatment [149,155]. 

## 11. Final Remarks

The role of HB-EGF in hematopoiesis is somehow surprising to us. HB-EGF is an ancient player on the field of evolution, where it plays a role in fundamental signaling mechanisms that are also involved in hematopoietic stem cell niches. It participates to such events in association with a variety of other factors and together they orchestrate the complex network of hematopoietic regulated differentiation. Due to the relevance of the bone marrow niches for oncogenesis, HB-EGF represents a molecule relevant to the biology of hematopoietic malignancies that develop primarily in the bone marrow microenvironment such as acute and chronic either myeloid or lymphoid leukemias, as well as multiple myeloma. It is possible that we can expect that the role of the bone marrow microenvironment is not a great deal different for malignancies arising in periphery and infiltrating the hematopoietic bone marrow secondarily. The relevance of these mechanisms is also supported by the promising results of targeted treatments in the preclinical and clinical setting. However, the very mechanisms, crosstalks and cell types fundamental for regulated differentiation in the hematopoietic system as well as their fine-tuning and global significance are far from being elucidated.
